# Divergence-Based Locally Weighted Ensemble Clustering with Dictionary Learning and *L*_2,1_-Norm

**DOI:** 10.3390/e24101324

**Published:** 2022-09-21

**Authors:** Jiaxuan Xu, Jiang Wu, Taiyong Li, Yang Nan

**Affiliations:** 1 School of Computing and Artificial Intelligence, Southwestern University of Finance and Economics, Chengdu 611130, China; 2 Department of Computer Science, Harbin Finance University, Harbin 150030, China

**Keywords:** clustering, ensemble clustering, *L*_2,1_-norm, similarity, subspace clustering, dictionary learning

## Abstract

Accurate clustering is a challenging task with unlabeled data. Ensemble clustering aims to combine sets of base clusterings to obtain a better and more stable clustering and has shown its ability to improve clustering accuracy. Dense representation ensemble clustering (DREC) and entropy-based locally weighted ensemble clustering (ELWEC) are two typical methods for ensemble clustering. However, DREC treats each microcluster equally and hence, ignores the differences between each microcluster, while ELWEC conducts clustering on clusters rather than microclusters and ignores the sample–cluster relationship. To address these issues, a divergence-based locally weighted ensemble clustering with dictionary learning (DLWECDL) is proposed in this paper. Specifically, the DLWECDL consists of four phases. First, the clusters from the base clustering are used to generate microclusters. Second, a Kullback–Leibler divergence-based ensemble-driven cluster index is used to measure the weight of each microcluster. With these weights, an ensemble clustering algorithm with dictionary learning and the $L_{2,1}$-norm is employed in the third phase. Meanwhile, the objective function is resolved by optimizing four subproblems and a similarity matrix is learned. Finally, a normalized cut (Ncut) is used to partition the similarity matrix and the ensemble clustering results are obtained. In this study, the proposed DLWECDL was validated on 20 widely used datasets and compared to some other state-of-the-art ensemble clustering methods. The experimental results demonstrated that the proposed DLWECDL is a very promising method for ensemble clustering.

## 1. Introduction

For a long time, clustering has been widely studied as an important technology for machine learning [1,2,3,4]. However, due to the lack of prior knowledge, i.e., pre-label training, the accuracy of clustering algorithms is much lower than that of supervised learning methods. Traditional single clustering methods, such as k-means, balanced iterative reducing and clustering using hierarchies (BIRCH), density-based spatial clustering of applications with noise (DBSCAN), etc., cannot usually achieve good clustering results for complex data [5,6]. Encouraged by the accuracy improvement effects of ensemble learning methods, many researchers have begun to study clustering ensemble algorithms. Clustering ensembles learn from multiple base clustering results to obtain consensus results, which can greatly improve the clustering accuracy without the need for prior knowledge [7,8,9,10,11,12].

The focuses of ensemble clustering methods are either the selection of base clustering or ensemble methods [13]. The selection of base clustering has two influences on the consensus results: accuracy and diversity. Higher accuracy usually leads to the lower diversity of the base clustering, while higher diversity results in the lower accuracy of the base clustering [14]. Therefore, balancing these two factors is key in the selection of base clustering. Ensemble methods aim to learn more robust consensus results by mining more effective information from the base clustering sets. Essentially, ensemble methods mine more inner information from the base clusterings. Although there are many robust ensemble methods, it is difficult to identify which ensemble method outperforms the others on a given dataset due to the randomness of the base clustering selection and the diversity of datasets.

Generally speaking, the most commonly used representative methods for mining this information from base clusterings include (1) co-association (CA) matrices, which represent the mutual relationships between samples in the base clustering sets, i.e., relationships at the sample level, (2) cluster–cluster (CC) matrices, which indicate the relationships between clusters in base clustering sets, i.e., relationships at the cluster level, and (3) sample–cluster matrices, which represent the relationships between samples and clusters in base clustering sets, i.e, relationships at the sample–cluster level. Both CA and CC matrices can be calculated using sample–cluster matrices. CA matrices reveal the probability that samples are of the same class. The larger the value of $X_{ij}$ in a CA matrix, the greater the possibility that the samples *i* and *j* are of the same class. Some methods aim to retain or learn reliable samples in CA matrices and then seek consensus results [10,14]. For example, Jia et al. proposed an effective self-enhancement framework for CA matrices to improve the ensemble clustering results, through which high-confidence information was extracted from base clusterings [15]. CC matrices reveal the similarities between clusters, which cannot be used for ensembles alone due to the lack of effective information, so it has to be combined with other valid information to perform accurate clustering. Therefore, some researchers have used CC matrices to calculate similarities and then mapped them as weights to CA matrices or sample–cluster matrices [11,16]. Sample–cluster matrices are the original matrices in base clustering sets and retain the most complete information in base clustering sets. Some methods choose to explore hidden information in the original matrices [11]. For example, based on sample–cluster matrices, the dense representation ensemble clustering (DREC) method introduces microcluster representation, reduces the amount of data, retains the effective information from sample–cluster matrices to the greatest extent and then performs dense representation clustering, which not only improves the time performance but also explores the hidden effective information to the greatest extent [13]. Huang et al. pointed out that the differences between microclusters also play important roles in ensemble clustering [17]. However, the DREC method ignores the differences between microclusters. Moreover, it does not reveal the underlying structures in sample–cluster matrices well. Entropy-based locally weighted ensemble clustering (ELWEC) has been demonstrated as being effective in improving clustering accuracy [18]. The key reason for this is the adoption of the idea of mapping entropy-based local weights to clustering. However, the ELWEC method measures the weights of clusters rather than microclusters and ignores sample–cluster relationships, thereby limiting the clustering performance to some extent. Very recently, the Markov process [19], a growing tree model [20], a low-rank tensor approximation [21] and an equivalence granularity [22] have been applied to ensemble clustering to achieve better clustering results.

Motivated by the above analysis, a divergence-based locally weighted ensemble clustering with dictionary learning (DLWECDL) is proposed in this paper. The idea of local weights was introduced to the DLWECDL. Different from the entropy-based local weights of clusters in ELWEC, this study used the divergence-based local weights of microclusters for ensemble clustering. Specifically, low-rank representation, the $L_{2,1}$-norm and dictionary learning were applied to design the objective function and the corresponding constraints. We used the augmented Lagrange multiplier (ALM) with alternating direction minimization (ADM) strategy for the optimization of the objective function. Extensive experiments on real datasets demonstrated the effectiveness of our proposed method.

The main contributions of this paper are summarized as follows:

(1) The proposal of a Kullback–Leibler divergence-based weighted method to better reveal relationships between clusters;
(2) The use of low-rank representation instead of dense representation to better explore hidden effective information and low-rank structures of original matrices;
(3) The application of the $L_{2,1}$-norm to noise to improve robustness;
(4) The introduction of adaptive dictionary learning to better learn low-rank structures;
(5) Extensive experiments to demonstrate that the proposed DLWECDL can significantly outperform other state-of-the-art approaches.

The rest of this paper is organized as follows. Section 2 reviews related works on ensemble clustering. The proposed ensemble clustering method is described in detail in Section 3. The experimental settings and results are analyzed and discussed in Section 4. Finally, Section 5 concludes the paper and provides our recommendations for future work.

## 2. Related Works

### 2.1. Ensemble Clustering

The goal of ensemble clustering is to find consensus results based on *M* base clusterings. To obtain good consensus results, two questions naturally arise. The first question is the selection of the base clusterings, which should not only ensure the diversity of the base clusterings but also the quality or accuracy of the base clusterings. Existing studies have proposed some methods that take into account the diversity and quality of base clusterings [23,24]. The second question is the ensemble method, which is roughly divided into two categories: similarity matrix-based learning and graph-based learning. Similarity matrices are the core problems in various clustering methods. In ensemble clustering, similarity matrices are obtained by exploring sample–sample, cluster–cluster and sample–cluster relationship matrices and then using spectral clustering to obtain the final clustering results.

Based on similarity matrices, our method follows a dense representation ensemble clustering framework, finds microclusters and then performs dense representation at the microcluster level. However, it does not work for microclusters that contain more samples. Therefore, we designed a local weight-based microcluster ensemble method and used a new low-rank representation clustering method. Inspired by the ALRR method [25], we introduced the $L_{2,1}$-norm and adaptive dictionary learning to the new low-rank representation method.

### 2.2. Microcluster Representatives

Our approach starts by finding microcluster representatives to simplify the problem. A sample–cluster matrix needs to be reconstructed before looking for these microcluster representatives.

**Figure 1:**
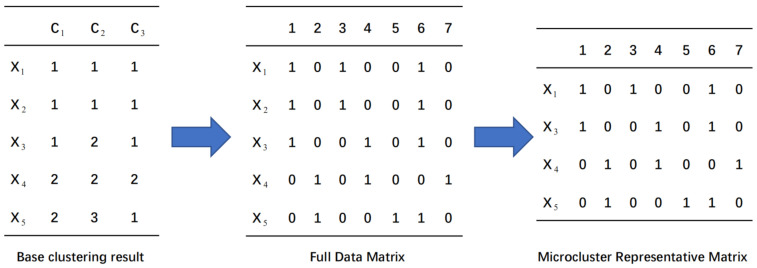
The reconstruction of a data matrix.

Figure 1 is an example that illustrates our definition of a microcluster, where $C_{i}$ is the *i*-th base clustering, $X_{j}$ represents the *j*-th sample and the numbers 1–7 in the heading of the full data matrix are the global renamed cluster IDs. We reconstructed the original base clustering results to obtain the full data matrix, in which we observed that the information in samples $X_{1}$ and $X_{2}$ was completely consistent. Therefore, we grouped $X_{1}$ and $X_{2}$ into the same microcluster and chose either $X_{1}$ or $X_{2}$ as the microcluster representative.

### 2.3. Information Entropy-Based Locally Weighted Method

The information entropy-based locally weighted method mainly explores the uncertainty of each cluster [18]. It introduces the concept of entropy to calculate the uncertainty of each cluster and then determines the weight of each cluster using a monotonically decreasing function. It forms results based on the more stable cluster, the smaller the uncertainty and the larger the weight. However, for similar clusters, it cannot guarantee that the final weights are consistent, even though the weights of completely different clusters may be consistent.

We used the locally weighted method for microclusters to calculate the weight of each cluster in each base clustering and then apply it to the microclusters. The weights were measured using the ensemble-driven cluster index (ECI).

Taking the cluster in the *i*-th base clustering $\pi_{i}$ as an example, the weights were calculated as follows:

(1) $$H\left(\pi_{i}\right)=\sum_{m=1}^{M}-\sum_{j=1}^{K}p\left(\pi_{i},\pi_{j}^{m}\right){log}_{2}p\left(\pi_{i},\pi_{j}^{m}\right)$$

(2) $$p\left(\pi_{i},\pi_{j}^{m}\right)=\frac{\left|\pi_{i}\cap \pi_{j}^{m}\right|}{\left|\pi_{i}\right|}$$

(3) $$ECI\left(\pi_{i}\right)=e^{-\frac{H\left(\pi_{i}\right)}{\theta M}}$$

where − represents the subtraction operation, θ is a control parameter and $|C_{i}|$ represents the number of samples in $C_{i}$. After obtaining the ECI weight of each cluster, we applied them to the selected representative microcluster matrix to obtain the final data matrix.

### 2.4. Dense Representation Ensemble Clustering

The concept of microclusters has been introduced into the DREC method. The scale of ensemble clustering problems is simplified using the “slim-down strategy” and then similarity matrices can be obtained using the dense representation method and the final result segmentation can be obtained by applying the Ncut algorithm. Because of the microclusters, the DREC method improves time efficiency and preserves more original information. However, the DREC method treats “shrunk” samples equally, which does not work for microcluster samples. At the same time, although the DREC method considers the influence of noise, it fails to consider the selection of the base clusterings, which also leads to the instability of the final results of the randomly selected base clustering integration.

## 3. Divergence-Based Locally Weighted Ensemble Clustering with Dictionary Learning (DLWECDL)

The goal of ensemble clustering is to learn consistent results based on *M* base clusterings. In ensemble clustering, the key is to explore the effective information in base clustering sets. The effective information in base clustering sets is hidden within three common manifestations, namely sample–sample relational representation, sample–cluster relational representation and cluster–cluster relational representation. We believe that good consensus results can be obtained when all of the valid information from the three representations can be fully utilized. Sample–cluster relationship matrices are key to linking these three representations because they can be used to calculate the remaining two representations. Therefore, we took the sample–cluster relational representation as the base representation and used it as the data matrix for our method. It was the original representation of our base clustering set.

### 3.1. Divergence-Based Locally Weighted Method

The information entropy-based locally weighted method mainly considers the uncertainty between clusters.We introduced the Kullback–Leibler (KL) divergence, which is widely used to measure the differences between distributions. When distributions are exactly the same, the KL divergence is 0. Considering the good performance of KL divergence in some clustering methods over recent years, we introduced KL divergence as a measure of local weights. Since $p\left(\pi_{i},\pi_{j}^{m}\right)$ and $p\left(\pi_{j}^{m},\pi_{i}\right)$ are not clear probabilistic interpretations, the KL divergence results here were not guaranteed to always be greater than 0. After obtaining the KL divergence, we used the ECI entropy mapping function to obtain the new KL divergence weights.

(4) $$KL\left(\pi_{i}\right)=\sum_{m=1}^{M}-\sum_{j=1}^{K}p\left(\pi_{i},\pi_{j}^{m}\right){log}_{2}\frac{p(\pi_{i},\pi_{j}^{m})}{p(\pi_{j}^{m},\pi_{i})}$$

**Figure 2:**
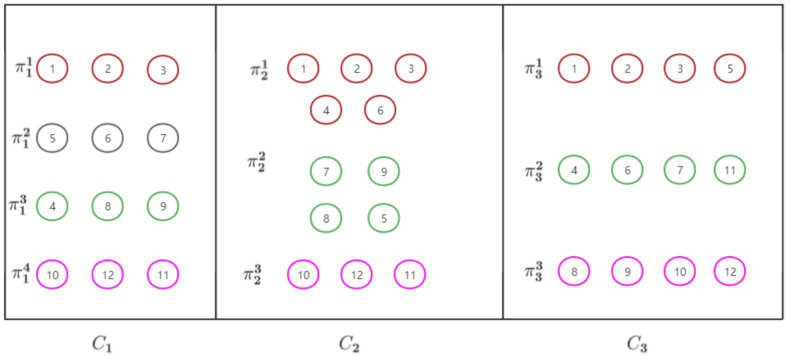
An example of the entropy and divergence value calculations.

To better illustrate the advantages of KL divergence weighting, an example is presented in Figure 2, where $C_{i}$ represents the *i*-th base clustering result, $\pi_{i}^{j}$ denotes the *j*-th cluster in the *i*-th base clustering and the numbers 1–12 in the circles are the numbers of the samples. As shown in Table 1, we compared the results of the inter-cluster entropy calculation and the KL divergence calculation, where *R* represents the ratio of the maximum number of samples in the stable subsets to the number of samples that were contained in the clusters. For example, Samples 1, 2 and 3 were assigned to $\pi_{1}^{1}$, $\pi_{2}^{1}$ and $\pi_{3}^{1}$ in the base clusterings $C_{1}$, $C_{2}$ and $C_{3}$, respectively. The three samples were classified into the same class in different base clustering results. This meant that the most stable subsets of $\pi_{1}^{1}$, $\pi_{2}^{1}$ and $\pi_{3}^{1}$ were Sample 1, Sample 2 and Sample 3, respectively. Therefore, the R values for clusters $\pi_{1}^{1}$, $\pi_{2}^{1}$ and $\pi_{3}^{1}$ were $\frac{3}{3}=1$, $\frac{3}{5}=0.6$ and $\frac{3}{4}=0.75$, respectively. It can be observed from Table 1 that the *R* values of $\pi_{1}^{3}$, $\pi_{1}^{4}$ and $\pi_{2}^{3}$ were consistent but the entropy values were quite different. This led to inconsistent weights. The same situation occurred in $\pi_{2}^{2}$ and $\pi_{3}^{2}$. The KL divergence method reduced the gaps between clusters with the same R values as much as possible so that the weights were as consistent as possible.

### 3.2. $L_{2,1}$-Norm Subspace Clustering of Adaptive Dictionaries

After obtaining the final data matrix, we developed a new subspace clustering method. Unlike dense representation, we explored similarity matrices using low-rank representation, which incorporated an adaptive dictionary learning strategy and employed a new regularization term, i.e., the $L_{2,1}$-norm.

The original low-rank subspace clustering that could explore similarity matrices was formulated as follows:

(5) $$\begin{array}{c} \min_{Z,E}{∥Z∥}_{*}+\lambda {∥E∥}_{2,1}\\ \;s.t.\;X=DZ+E \end{array}$$

where λ is a regularization parameter, *X* represents the data matrix, *D* is the dictionary, *Z* is the low-rank representation coefficient matrix, *E* is the noise and ${\left||.|\right|}_{*}$ and ${\left||.|\right|}_{2,1}$ represent the nuclear norm and the $L_{2,1}$-norm of the matrix, respectively. The original low-rank representation method the data *X* as a dictionary *D*. On this basis, many low-rank representation subspace clustering algorithms have been further proposed and the adaptive dictionary learning low-rank representation [25] problem can be formulated as follows:

(6) $$\begin{array}{c} \min_{Z,E}{∥Z∥}_{*}+\lambda {∥X-DZ∥}_{F}^{2}\\ \;s.t.\;DD^{T}=I_{d} \end{array}$$

where ${\left||.|\right|}_{F}$ is the famous Frobenius norm, which was used here for computational convenience because many closed-form solutions that are based on this norm can greatly improve time efficiency. In order to eliminate the arbitrary scaling factor in the process of dictionary learning, *D* and *X* were replaced by $P^{T}X$. To take into account the advantages of dictionary learning and noise immunity, our method was formulated as follows:

(7) $$\begin{array}{c} \min_{Z,P,E}{∥Z∥}_{*}+\lambda {∥E∥}_{2,1},\\ \;s.t.\;X=P^{T}XZ+E\;P^{T}XX^{T}P=I_{d} \end{array}$$

where *P* denotes a low-dimensional projection matrix and $I_{d}$ is the identity matrix. The proposed method not only retains dictionary learning in low-rank representation, i.e., learning better and more orthogonal dictionaries, but also adopts the $L_{2,1}$-norm to make it more robust to noise. A widely accepted theory is that high-dimensional data are determined by low-dimensional structures. The low-rank matrix *Z* that was obtained according to the objective function contained the angle information between the data samples. We performed SVD decomposition on the low-rank matrix *Z* and obtained $H=U\Sigma^{\frac{1}{2}}$. We then used *H* to obtain the final similarity matrix *W*.

**Figure 3:**
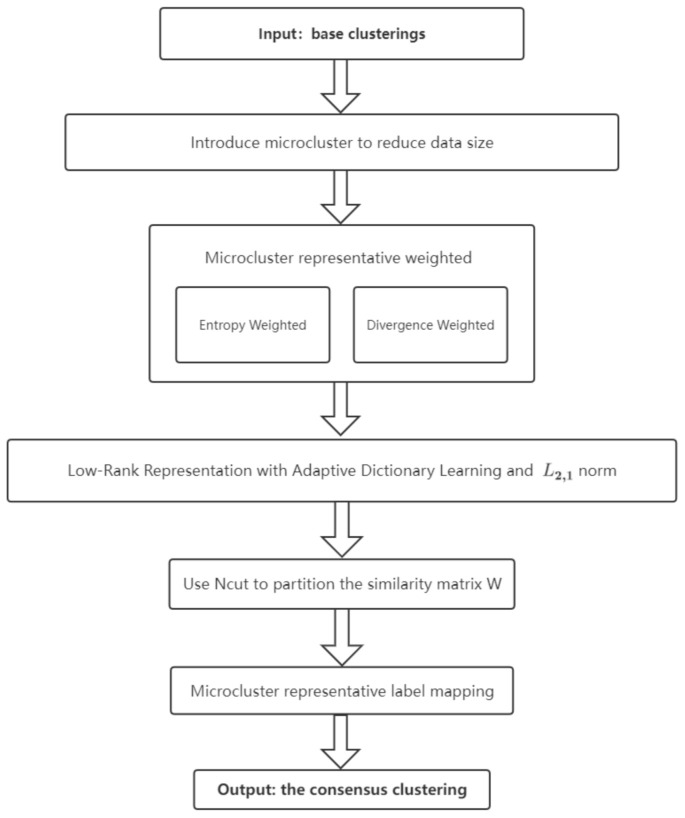
A flowchart of the proposed DLWECDL.

The detailed steps of the proposed DLWECDL are described in Algorithm 1, a flowchart for which is also shown in Figure 3. It should be noted that α in Algorithm 1 is a positive integer parameter and $h_{i}$ and $h_{j}$ are the *i*-th and *j*-th rows of matrix H, respectively.
**Algorithm 1:**Divergence-based locally weighted ensemble clustering with dictionary learning (DLWECDL).**Input**: *M* base clustering, $\left[C_{1},C_{2},\cdots ,C_{m}\right]$.**Output**: Consensus clustering result *S*1. Reconstruct the data matrix to obtain a microcluster representative matrix.2. Calculate the local divergence weight and local entropy weight and weigh the microcluster representative matrix.3. Learn low-rank structures *Z* by Low-rank representation with adaptive dictionary learning and the $L_{2,1}$-norm.4. Calculate *H* by SVD decomposition of *Z*. Calculate similarity matrix *W* by *H*.                $H=U\Sigma^{\frac{1}{2}}$, $Z=SVD(U\Sigma V^{T}$), ${\left[W\right]}_{ij}$ = ${\left(\frac{h_{i}h_{j}^{T}}{∥h_{i}{∥}_{2}{∥h_{j}∥}_{2}}\right)}^{2\alpha }$5. Perform Ncut to partition the similarity matrix *W*.6. Obtain consensus result S by microcluster representative label mapping.

As shown in Figure 3, DLWECDL first introduces microclusters to reduce the amount of data, which reduces data redundancy and improves time efficiency. Then, DLWECDL performs local weighting on the simplified dataset. Two weighting methods, namely entropy-based and KL divergence-based weighting, are used to better represent the microclusters. Theoretically, the entropy-based weighting method focuses more on the uncertainty of the clusters themselves while the KL divergence-based method focuses more on the relative uncertainty, i.e., the differences between clusters. This also means that datasets with more diverse base clusterings may be more suitable for the KL divergence-based weighting method. The third step uses low-rank representation with dictionary learning and the $L_{2,1}$-norm to explore deep structures. After using the Ncut method to partition the data, the labels of the reduced dataset need to be mapped to the full dataset because of the introduction of the microclusters.

**Figure 4:**
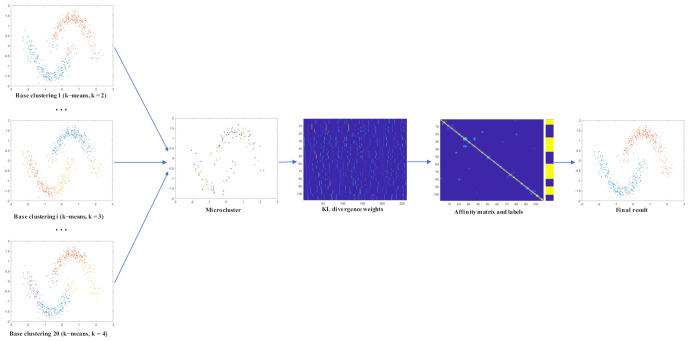
An example on a synthetic dataset.

To demonstrate the feasibility and effectiveness of the proposed algorithm more intuitively, an example on a 2D synthetic dataset is presented in Figure 4. In the example, k-means clustering algorithms with different *k*s were performed 20 times. Their outputs were used to generate the microclusters, from which a matrix of the KL divergence weights was obtained. Then, low-rank representation with adaptive dictionary learning and the $L_{2,1}$-norm was applied to the weighted matrix to obtain an affinity matrix and the corresponding labels for the microclusters. Finally, the labels were mapped to obtain the final results of the proposed DLWECDL. In Figure 4, the microclusters, KL divergence weights, affinity matrix and labels are the intermediate data of the proposed DLWECDL.

### 3.3. Optimization Method

For Problem (7), we employed the augmented Lagrange multiplier (ALM) with alternating direction minimization (ADM) strategy for optimization [26]. The auxiliary variable *J* needed to be introduced here. The augmented Lagrangian function is as follows:

(8) $$\begin{array}{c} L={∥J∥}_{*}+\lambda {∥E∥}_{2,1}+\mathrm{tr}\left(Y_{1}^{T}\left(X-P^{T}XZ-E\right)\right)+\mathrm{tr}\left(Y_{2}^{T}\left(Z-J\right)\right)+\\ \frac{\mu }{2}\left({∥X-P^{T}XZ-E∥}_{F}^{2}+{∥Z-J∥}_{F}^{2}\right),\\ \;s.t.\;P^{T}XX^{T}P=I_{d} \end{array}$$

where $Y_{1}$ and $Y_{2}$ are Lagrange multipliers and μ is a penalty parameter. According to the ADM strategy [26], we divided the objective into several subproblems that could be efficiently optimized.

#### 3.3.1. Subproblem *J*

To update *J*, we needed to solve the following problem:

(9) $$J^{*}={\mathrm{argmin}}_{J}\frac{1}{\mu }{∥J∥}_{*}+\frac{1}{2}{∥J-\left(Z+Y_{2}/\mu \right)∥}_{F}^{2}$$

Problem (9) had a popular closed-form solution, which was solved using SVD decomposition. It was consistent with the first solution of the LRR method.

#### 3.3.2. Subproblem *Z*

To update *Z*, we needed to solve the following problem:

(10) $$Z^{*}={\mathrm{argmin}}_{Z}\;\;tr\left(Y_{1}^{T}\left(X-P^{T}XZ-E\right)\right)+tr\left(Y_{2}^{T}\left(Z-J\right)\right)+\frac{\mu }{2}(||X-P^{T}XZ-E{\left|\right|}_{F}^{2}+||Z-J{\left|\right|}_{F}^{2})$$

Since Problem (9) was unconstrained, we could take the derivative of *Z* directly. We obtained the derivation result of Problem (10) as follows:

(11) $$\frac{\partial L}{\partial Z}=-X^{T}PY_{1}+Y_{2}+\frac{\mu }{2}\left(-2X^{T}PX+{2X}^{T}PP^{T}XZ+2X^{T}PE+Z-J\right)$$

Let $\frac{\partial L}{\partial Z}=0$, then we could obtain the result of *Z* as follows:

(12) $$Z^{*}={\left(X^{T}PP^{T}X+I\right)}^{-1}\left(\frac{X^{T}PY_{1}-Y_{2}}{\mu }+J+X^{T}P\left(X-E\right)\right)$$

#### 3.3.3. Subproblem *E*

To update *E*, we needed to solve the following problem:

(13) $$E^{*}={\mathrm{argmin}}_{E}\;\frac{\lambda }{\mu }{\left||E|\right|}_{2,1}+\frac{1}{2}||E-\left(X-P^{T}XZ+Y_{1}/\mu \right){\left|\right|}_{F}^{2}$$

As with Problem (9), Problem (13) also had a closed-form solution. We calculated *E* using Lemma 1.

**Lemma** **1.** *Let $Q=[q_{1},q_{2},\cdots ,q_{i},\cdots ]$ be a given matrix. When the optimal solution to*

(14) $$\min_{W}{\lambda ∥W∥}_{2,1}+\frac{1}{2}{∥W-Q∥}_{F}^{2}$$

is $W^{*}$, then the i−th column of $W^{*}$ is

(15) $${\left[W^{*}\right]}_{:,i}=\left\{\begin{array}{cc} \frac{{∥q_{i}∥}_{2}-\lambda }{{∥q_{i}∥}_{2}}q_{i} & \;\mathrm{if}\;\lambda <{∥q_{i}∥}_{2}\\ 0 & \;otherwise\; \end{array}\right.$$

#### 3.3.4. Subproblem *P*

To update *P*, we needed to solve the following problem:

(16) $$P^{*}={\mathrm{argmin}}_{P}\;\;tr\left(Y_{1}^{T}\left(X-P^{T}XZ-E\right)\right)+\frac{\mu }{2}\left|\right|X-P^{T}XZ-E{\left|\right|}_{F}^{2}\;s.t.\;P^{T}XX^{T}P=I_{d}$$

Considering that Problem (16) was a constrained problem, we introduced Lemma 2 to solve it.

**Lemma** **2.** *Given the objective function $\min_{R}{∥Q-GR∥}_{F}^{2}\cdot \;s.t.\;R^{T}R=RR^{T}=I$, the optimal solution is $R=UV^{T}$, where U and V are the left and right singular values of the SVD decomposition of $G^{T}Q$, respectively.*

We transformed Problem (16) to obtain the following results:

$$P^{*}={\mathrm{argmin}}_{P}\;\;\frac{\mu }{2}\left|\right|X-P^{T}XZ-E+Y_{1}/\mu {\left|\right|}_{F}^{2}\;\;\;s.t.\;P^{T}XX^{T}P=I_{d}$$

Going one step further:

$$P^{*}={\mathrm{argmin}}_{P}\frac{\mu }{2}\left|\right|{\left(X+Y_{1}/\mu -E\right)}^{T}-Z^{T}X^{T}P{\left|\right|}_{F}^{2}\;\;s.t.\;P^{T}XX^{T}P=I_{d}$$

Let $X^{T}P=R$, then according to Lemma 2, we could obtain the equation $X^{T}P=UV^{T}$. Then, we only needed to calculate the inverse of the data matrix to obtain the solution to Problem (16): $P={\left(X^{T}\right)}^{-1}UV^{T}$.

The detailed optimization algorithm for DLWECDL is shown in Algorithm 2.
**Algorithm 2:**Optimization algorithm for DLWECDL.
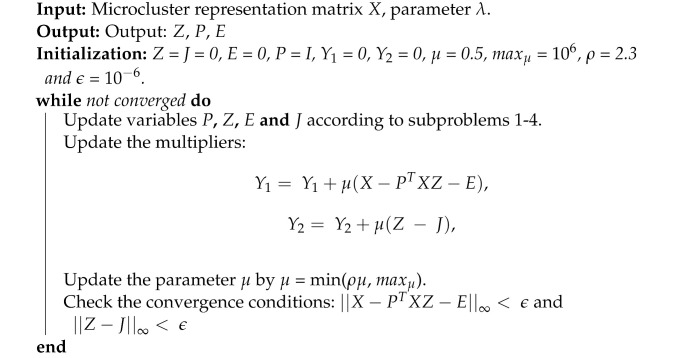


#### 3.3.5. Differences between Our Approach and Other Ensemble Clustering Methods

As mentioned in the Introduction, our method introduces the theory of microclusters in order to reduce the dataset size. The divergence weights are then calculated and applied to the microclusters. Finally, a low-rank representation is performed to obtain a similarity matrix. Compared to other existing advanced methods, our method has a great number of differences and advantages, mainly in the following aspects:

(1) Differences in the data matrix. Some methods perform ensemble algorithms based on co-association (CA) matrices [10,27], but CA matrices focus on instance-level relationships and ignore the relationships between clusters. Our method is based on instance–cluster data matrices, although the DREC [13], PTA-CL [17] and CESHL [11] methods also use data matrices that are similar to ours. Among these methods, CESHL does not introduce microclusters and its time efficiency is low. DREC fails to consider the differences between microclusters. Our method makes up for these shortcomings. It is worth pointing out that although the PTA-CL method considers the differences between microclusters, it does not explore their deep structures.
(2) Differences in the weighting methods. The LWEC method is based on the entropy-based weighting method [18]. As shown in Section 3.1, the entropy-based weighted method cannot solve the problem of consistent weights among the similar clusters. Therefore, our method uses KL divergence-based weighting to alleviate this contradiction to a certain extent. Some other weighting methods focus on cluster-level similarities and then map these similarities to the instance level [16].
(3) Differences in the low-rank representation. The existing low-rank representation-based ensemble methods all treat the original data directly as a dictionary [28,29]. Considering that good dictionaries are crucial to the learning of similarity matrices, our method uses novel low-rank representation with dictionary learning constraints.

## 4. Experiments

### 4.1. Datasets and Evaluation Methods

In this section, we present the setup and results of our extensive experiments to validate the proposed algorithm on 20 real datasets. Information about the datasets is listed in Table 2.

Although there are various metrics for evaluating clustering performance, we chose three of them, namely accuracy (ACC), normalized mutual information (NMI) and adjusted rand index (ARI), to evaluate the proposed approach because of their simplicity, popularity and robustness to changes in labeling [18,30].

ACC is the score that is obtained by matching ground truth labels. Since the labels that are assigned by clustering methods may be inconsistent with the ground truth labels, the Hungarian algorithm is generally used for label alignment when calculating ACC, which can be formulated as follows:

(17) $$ACC=\max_{f}\frac{1}{n}\sum_{j=1}^{n}\delta \left(y_{j},f\left(\pi \left(x_{j}\right)\right)\right),$$

where $y_{j}$ represents the ground truth labels and $\delta (y_{j},f\left(\pi \left(x_{j}\right)\right))=1$ when $y_{j}=f(\pi \left(x_{j}\right)$ and $\delta (y_{j},f\left(\pi \left(x_{j}\right)\right))=0$ otherwise.

As a measure of mutual information entropy that indicates the clustering results and the ground truth labels [31], NMI is defined as follows:

(18) $$NMI=\frac{\sum_{p}\sum_{q}n_{p,q}\log\left(\frac{n\cdot n_{p,q}}{n_{p}\cdot n_{q}}\right)}{\sqrt{\sum_{p}n_{p}\log\frac{n_{p}}{n}\left(\sum_{q}n_{q}\log\frac{n_{q}}{n}\right)}},$$

where the cluster $c_{p}$ in the clustering results and the cluster $c_{q}$ in the ground truth labels contain $n_{p}$ and $n_{q}$ instances, respectively.

ARI is an improved version of the rand index (RI) that can reflect the degree of overlap between clustering results and ground truth labels [32], which can be defined as follows:

(19) $$ARI=\frac{\left(\begin{array}{c} N\\ 2 \end{array}\right)\sum_{i=1}^{k}\sum_{j=1}^{k^{\prime }}\left(\begin{array}{c} N_{i,j}\\ 2 \end{array}\right)-\left[\sum_{j=1}^{k}\left(\begin{array}{c} N_{i}^{c}\\ 2 \end{array}\right)\sum_{j=1}^{k^{\prime }}\left(\begin{array}{c} N_{j}^{p}\\ 2 \end{array}\right)\right]}{\frac{1}{2}\left(\begin{array}{c} N\\ 2 \end{array}\right)\left[\sum_{i=1}^{k}\left(\begin{array}{c} N_{i}^{c}\\ 2 \end{array}\right)+\sum_{j=1}^{k^{\prime }}\left(\begin{array}{c} N_{j}^{p}\\ 2 \end{array}\right)\right]-\left[\sum_{i=1}^{k}\left(\begin{array}{c} N_{i}^{c}\\ 2 \end{array}\right)\sum_{j=1}^{k^{\prime }}\left(\begin{array}{c} N_{j}^{p}\\ 2 \end{array}\right)\right]},$$

where the clustering results and the ground truth labels contain *k* and $k^{{}^{\prime }}$ clusters, respectively, $N_{i,j}$ is the number of common instances in cluster $c_{i}$ in the clustering results and cluster $p_{j}$ in the ground truth labels and $N_{i}^{c}$ and $N_{j}^{p}$ are the numbers of instances in clusters $c_{i}$ and $p_{j}$, respectively.

The definitions of these three evaluation indicators show that the greater the indicator values, the better the method.

### 4.2. Experimental Settings

Each of the selected datasets contained 100 base clustering results, from which we randomly selected 20 to evaluate the ensemble clustering in each run. There were two main hyperparameters in the proposed approach, namely θ in (3) and λ in Problem (7). We used the grid search method to optimize the hyperparameters with all of the data in each dataset using the set of {0.2:0.1:2} for θ and {0.01,0.1,1,10,100,200,500} for λ. Note that these hyperparameters could also be optimized using evolutionary algorithms, as in many practical applications [33,34,35,36]. Additionally, the true number of classes in each dataset was also used as the input of the proposed approach. For each dataset, we ran the experiments 20 times and then reported the average results.

### 4.3. Experimental Results

We carried out a large number of repeated experiments and obtained average results, according to the optimal parameter range. We also compared our method to the following models:

- **DREC** [13], which introduces microclusters to reduce the amount of data and is a dense representation-based method;
- **LWGP, LWEA** [18], which both use locally weighted methods (LWGP is based on graph partitioning and LWEA is based on hierarchical clustering);
- **MCLA** [37], which is a clustering ensemble method that is based on hypergraph partitioning;
- **PTA-CL** [17], which introduces microclusters, explores probabilistic trajectories based on random walks and then uses complete-linkage hierarchical agglomerative clustering;
- **CESHL** [11], which is a clustering ensemble method for structured hypergraph learning;
- **SPCE** [10], which introduces a self-paced learning method to learn consensus results from base clusterings;
- **TRCE** [27], which is a multi-graph learning clustering ensemble method that considers tri-level robustness.

Note that the proposed DLWECDL used divergence-based local weights for ensemble clustering. We also replaced the divergence-based local weights in DLWECDL with entropy-based local weights but kept the other components unchanged in another algorithm, called ELWECDL, for comparison.

The NMI values of the proposed DLWECDL method and the other selected methods are listed in Table 3, where the best and second best values are shown in bold. From this table, it can be seen that the proposed DLWECDL method achieved the best or second best result in 16 out of the 20 cases, followed by the SPCE and ELWECDL methods (best or second best in 8 out of the 20 cases). TRCE achieved the best or second best result three times, meaning it ranked fourth among the ten methods. DREC, LWGP, LWEA, PTA-CL and CESHL performed so poorly that they all only achieved the best or second best result once. MCLA did not achieve the best or second best value for any of the 20 datasets. On average, DLWECDL and ELWECDL improved the NMI values by 7.36% and 6.12%, respectively, compared to the other eight ensemble clustering models. Therefore, these results demonstrated that the proposed DLWECDL significantly outperformed the other selected ensemble clustering methods in terms of NMI.

The ARI values of the ensemble clustering methods that are shown in Table 4 offered the following findings: (1) the DLWECDL method achieved the best or second best results 17 times, meaning that it ranked first among all of the ensemble clustering methods once again; (2) the DLWECDL method was followed by the ELWECDL method, which achieved the best or second best results for nine datasets; (3) the rest of the methods only achieved the best or second best values three times or less and specially, both MCLA and TRCE failed to achieve the best or second best values for any of the datasets; (4) on average, the ARI values of DLWECDL and ELWECDL improved by 15.11% and 12.49%, respectively, compared to the other models. These findings confirmed that the proposed DLWECDL method was superior to the other selected methods in terms of ARI.

**Figure 5:**
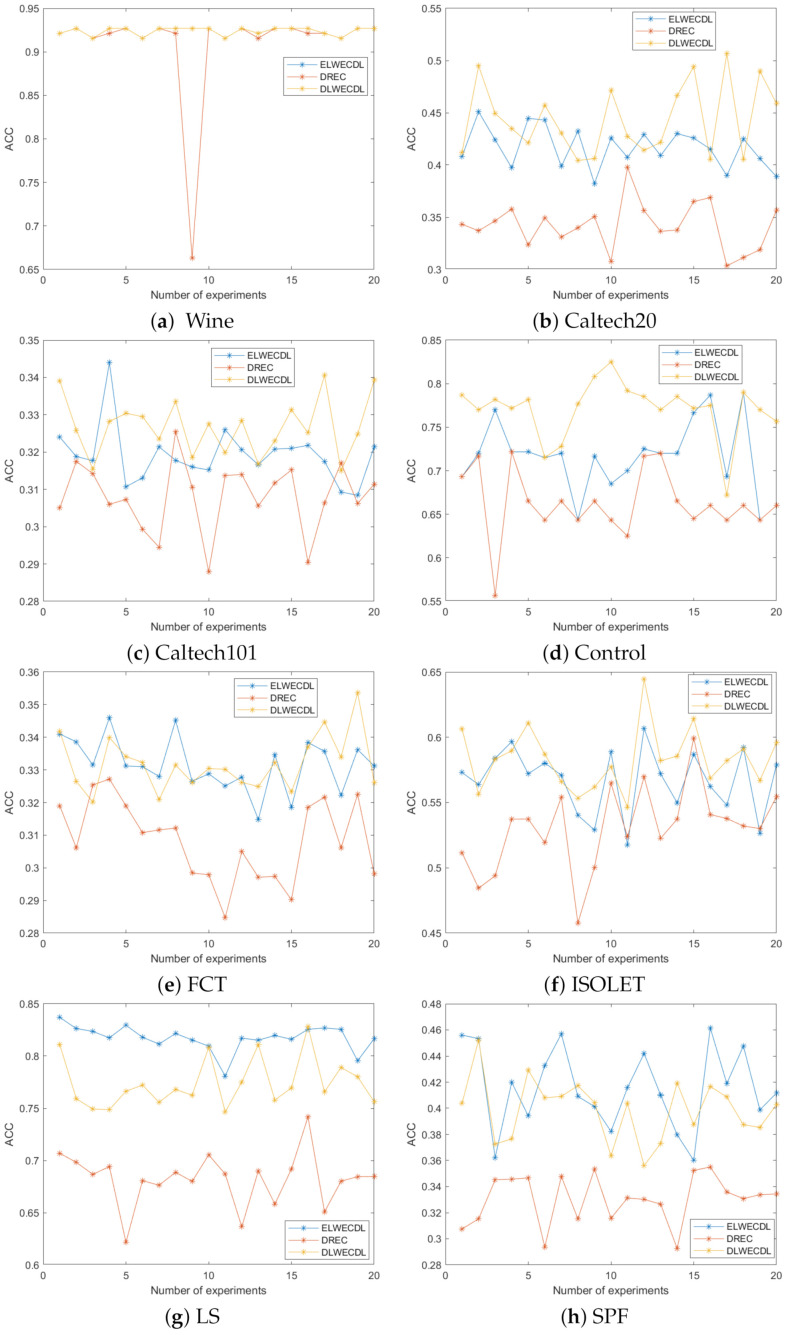
Our comparison of the accuracy of the three methods.

We further ran DREC, ELWECDL and DLWECDL on eight datasets (Wine, Caltech20, Caltech101, Control, FCT, ISOLET, LS and SPF). Each ensemble clustering method was run 20 times on each dataset and the accuracy values are plotted in Figure 5. We found that the ELWECDL and DLWECDL methods achieved much higher accuracy than the DREC method in almost all cases. Meanwhile, the DLWECDL method was advantageous over the ELWECDL method in most cases, which indicated that the divergence-based local weights were better than the entropy-based local weights for ensemble clustering.

### 4.4. Impact of Hyperparameters

**Figure 6:**
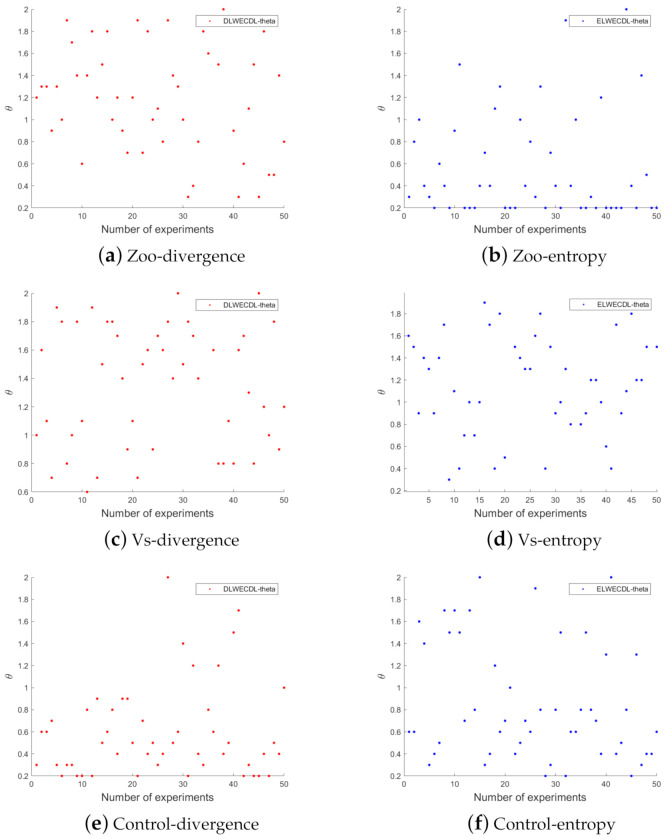
The weight parameter change diagrams that corresponded to the optimal results of a single repeated experiment.

For the proposed ensemble clustering algorithm, there are two main hyperparameters, i.e., λ in Problem (7) and θ in ECI. According to our extensive experiments, we found that λ had little effect on the final clustering results. The reason for this is the fact that low-rank structures are mainly explored using low-rank subspace clustering methods and ${∥Z∥}_{*}$ dominated Problem (7), as confirmed by Chen et al. [25]. For the weight parameter θ, we found that it had a large influence on the final results and that the optimal value of θ was related to the random selection of base clusterings in each experiment. According to our experience, the optimal weight parameter was 0.2–2. We selected some other datasets and repeated the experiments another 50 times. The θ values that corresponded to the maximum NMI values are shown in Figure 6.

As shown in Figure 6a, in the first run of the experiment on the Zoo dataset, the corresponding optimal θ value was 1.2, which became 1.3 in the second runt. In the subsequent experiment runs, θ did not have a fixed optimal value. The other datasets showed this same trend, which indicated that the parameter θ in our method was associated with the data matrix, i.e., we could not fix the weight parameter θ, even within the same dataset. This was mainly due to the problem of base clustering set selection.

### 4.5. Running Time

We compared the running time of the selected algorithms on 10 datasets, as shown in Table 5. As can be seen from the table, the time efficiency of the DLWECDL algorithm was not good because many iterations were performed while looking for low-rank representation. In order to reduce the number of iterations, we could adjust the learning rate, i.e., ρ, within an appropriate range, as long as the loss function was reasonably reduced. By adjusting the ρ value, we could control the number of iterations at less than 10, thereby improving the time performance of the algorithm. As can be seen from the table, after we increased the ρ value, the running time of DLWECDL became less than that of DREC [13].

### 4.6. Discussion

As analyzed in Section 3.3.5, our method is different from the other selected ensemble clustering methods in several aspects. Among them, the CESHL, DREC, PTA-CL and PTGP methods are based on the same data matrix as ours, while TRCE and SPCE are based on CA matrices. DREC, PTA-CL and PTGP all introduce microclusters to reduce the amount of data, while CESHL uses all data matrices directly. The superiority of our method over these methods mainly stems from the idea of the weighting and low-rank representation methods.

The KL divergence-based weighting method measures the differences between clusters, which alleviates the problem of the significant weight differences between similar clusters in ELWEC. Currently, DREC treats all microclusters equally and fails to consider the differences between microclusters. Although PTA-CL, PTGP and CESHL consider the differences between microclusters or clusters, none of them apply low-rank representation, i.e., they offer an insufficient exploration of the underlying information within data matrices. Moreover, CESHL is limited by the scale of the data, which leads to lower time efficiency.

Clustering ensemble method based on low-rank representation, such as RSEC, NRSEC, etc., are based on CA matrices and focus on instance-level relationships. They also all use the original data directly, i.e., the CA matrices, as dictionaries, although the $L_{2,1}$-norm is applied to consider the influence of noise. In general, the advantages of dictionary learning are more obvious.

## 5. Conclusions

In this paper, we proposed a new weighting method and a new low-rank representation method with adaptive dictionary learning. The new weighting method was able to mine more effective cluster–cluster relationships. We mapped these inter-cluster relationships into a representative microcluster matrix, i.e., we used the microcluster–cluster matrix as a new data matrix, and added new effective information on the basis of retaining the original matrix information to the greatest possible extent. Furthermore, methods based on low-rank representation with adaptive dictionary learning have been shown to be effective and we used a more reasonable $L_{2,1}$-norm to enhance robustness. Our experimental results demonstrated the effectiveness of our proposed method. On average, the proposed DLWECDL improved the NMI and ARI values by 7.36% and 15.11%, respectively, compared to the other selected SOTA ensemble clustering models. However, due to the influence of the random selection of base clusterings, we could not obtain a fixed optimal weight parameter that matched all possible base clustering combinations, even within the same dataset. Through our extensive experiments, we obtained an empirical range of weight parameters. The selection of the optimal combination of base clusterings within a dataset to obtain a pre-determined optimal weight parameter is our next research direction.

## Figures and Tables

**Table 1:** The entropy values versus the KL divergence values.

Cluster	$\pi_{1}^{1}$	$\pi_{1}^{2}$	$\pi_{1}^{3}$	$\pi_{1}^{4}$	$\pi_{2}^{1}$	$\pi_{2}^{2}$	$\pi_{2}^{3}$	$\pi_{3}^{1}$	$\pi_{3}^{2}$	$\pi_{3}^{3}$
R	1	0.33	0.67	0.67	0.6	0.5	0.67	0.75	0.5	0.25
Entropy	0	1.837	1.837	0.918	2.342	2.500	0.918	1.623	3.000	2.000
Divergence	0	−0.650	−0.650	−0.288	0.734	0.288	−0.288	0.120	0.248	0.432

**Table 2:** The characteristics of the datasets.

Dataset	Instances	Features	Classes	Dataset	Instances	Features	Classes
Zoo	101	16	7	ISOLET	7797	617	26
Control	600	60	6	MNIST	5000	784	10
Segment	2310	18	7	ODR	5620	64	10
MnistData_05	3495	653	10	Semeion	1593	256	10
Binalpha	1404	320	36	SPF	1941	27	7
MnistData_10	6996	688	10	Texture	5500	40	11
Caltech101	8671	784	101	VS	846	18	4
Caltech20	2386	30,000	20	Wine	178	13	3
FCT	3780	54	7	MF	2000	649	10
IS	2310	19	7	LS	6435	36	6

**Table 3:** Our comparison of the proposed method to the other selected methods, according to NMI.

Datset	DREC [13]	LWGP [18]	LWEA [18]	MCLA [37]	PTA-CL [17]	CESHL [11]	SPCE [10]	TRCE [27]	ELWECDL	DLWECDL
VS	0.1487	0.1320	0.1330	0.1472	0.1037	0.1444	**0.1655**	0.1368	0.1527	**0.1592**
Texture	0.7693	0.7430	0.7780	0.7220	0.6963	0.7552	**0.7850**	0.7610	**0.7942**	0.7778
SPF	0.1490	0.1520	0.1510	0.1350	0.0808	0.1398	**0.2120**	0.1330	0.1726	**0.1853**
Semeion	0.6563	0.6420	0.6550	0.5603	**0.6695**	0.6584	0.6256	0.6387	0.6645	**0.6646**
ODR	0.7442	0.8160	**0.8290**	0.6220	0.6172	0.8234	0.8193	0.8225	0.8230	**0.8282**
ISOLET	0.7168	0.7430	0.7450	0.6798	0.7018	0.7491	0.7358	**0.7502**	0.7475	**0.7545**
MNIST	0.6121	0.6350	0.6460	0.5141	0.6102	0.6252	0.6006	0.6309	**0.6762**	**0.6740**
FCT	0.2320	0.2000	0.2310	0.1730	0.2452	0.2015	**0.2720**	0.1980	**0.2593**	0.2574
MF	0.6553	0.6820	0.6590	0.6170	0.6290	0.6576	0.6737	0.6500	**0.6933**	**0.6886**
LS	0.6257	0.6440	0.6160	0.5500	0.5950	0.6412	0.5660	**0.6620**	**0.6699**	0.6425
Control	0.7215	0.6840	0.6850	0.7181	0.5963	0.6789	**0.7307**	0.7054	0.7166	**0.7526**
Wine	0.7523	0.7607	0.7630	N/A	N/A	0.7653	0.7645	**0.7688**	0.7679	**0.7682**
IS	0.6433	0.6290	0.6210	0.6367	0.6225	0.6288	0.5904	0.6152	**0.6597**	**0.6682**
Binalpha	0.5888	0.5502	0.5557	0.5824	0.5651	0.5439	**0.6068**	0.5953	0.5963	**0.6068**
Caltech101	0.5407	0.5327	N/A	0.5221	0.5359	N/A	N/A	N/A	**0.5486**	**0.5559**
Caltech20	0.4204	0.4300	0.4520	0.3844	0.4181	0.4345	**0.4600**	0.4590	0.4490	**0.4630**
Mnist_DATA_05	0.5059	**0.5065**	0.4975	0.4699	0.4987	0.4997	0.5039	0.5010	0.5017	**0.5062**
Mnist_DATA_10	**0.5016**	0.4817	0.4637	0.4876	0.5004	0.4963	0.4821	0.4988	0.4924	**0.5020**
ZOO	0.8312	0.8468	0.8036	0.7860	0.7773	**0.8869**	**0.8981**	0.8704	0.8635	0.8652
Segment	0.5967	0.5889	0.5990	0.5944	0.5894	0.6061	0.5993	0.6096	**0.6066**	**0.6188**

**Table 4:** Our comparison of the proposed method to the other selected methods, according to ARI.

Datset	DREC [13]	LWGP [18]	LWEA [18]	MCLA [37]	PTA-CL [17]	CESHL [11]	SPCE [10]	TRCE [27]	ELWECDL	DLWECDL
VS	**0.1248**	0.0970	0.1160	0.1189	0.0775	0.1235	0.1004	0.1127	0.1134	**0.1249**
Texture	0.6219	0.6200	**0.6890**	0.5970	0.5774	0.6400	0.5780	0.6210	**0.7116**	0.6807
SPF	**0.1110**	0.0830	0.0840	0.0874	0.0449	0.0659	0.0880	0.0590	0.1098	**0.1191**
Semeion	0.5468	0.5200	0.5390	0.4250	**0.5625**	0.5377	0.4742	0.5013	0.5427	**0.5488**
ODR	0.7675	0.7630	0.7820	0.6495	0.6647	0.7659	**0.7833**	0.7677	0.7789	**0.7832**
ISOLET	0.4781	0.5180	**0.5550**	0.4438	0.4675	0.5360	0.4788	0.5225	0.5396	**0.5641**
MNIST	0.4828	0.5120	0.5500	0.3800	0.5145	0.4894	0.4676	0.4879	**0.5884**	**0.5814**
FCT	0.1236	0.1170	0.1290	0.0933	0.1548	0.1242	0.1130	0.0950	**0.1754**	**0.1769**
MF	0.5284	0.5620	0.5250	0.5430	N/A	0.5217	0.5346	0.5210	**0.5804**	**0.5707**
LS	0.5463	0.5800	0.5680	0.4960	0.4520	0.5819	0.4750	0.5880	**0.6913**	**0.6448**
Control	0.5905	0.5415	0.5480	0.5847	0.4782	0.5580	**0.5963**	0.5675	0.5884	**0.6328**
Wine	0.7577	**0.7760**	0.7740	N/A	N/A	0.7710	0.7756	0.7753	0.7753	**0.7760**
IS	0.5370	0.5290	0.5220	0.5305	0.5165	0.5348	0.4803	0.4996	**0.5670**	**0.5680**
Binalpha	0.2988	0.3000	0.2890	0.2940	0.2807	0.2607	0.2816	0.2976	**0.3136**	**0.3227**
Caltech101	0.2823	0.2447	N/A	0.2551	**0.3054**	N/A	N/A	N/A	0.3044	**0.3332**
Caltech20	0.3098	0.2670	0.3520	0.2730	0.3046	**0.3672**	0.3170	0.2370	0.3386	**0.3719**
Mnist_DATA_05	0.3893	0.3750	0.3907	0.3273	0.3784	**0.3948**	0.3832	0.3690	**0.3923**	0.3880
Mnist_DATA_10	**0.4014**	0.3706	0.3883	0.3800	**0.4136**	0.3932	0.3778	0.3876	0.3894	0.3977
ZOO	0.8203	0.7935	0.7054	0.6715	0.6716	0.9253	**0.9473**	0.8790	0.8617	**0.8840**
Segment	0.4928	0.4619	0.4919	0.4881	0.4390	0.4994	0.4967	0.4919	**0.5048**	**0.5154**

**Table 5:** Our comparison of the time performance for smaller ρ values.

	HGBF [38]	SEC [39]	PTGP [17]	PTA-AL [17]	MCLA [37]	LWGP [18]	DREC [13]	DLWECDL (ρ=2.3)	DLWECDL (ρ=3.5)
Caltech20	1.2648	8.2952	0.1628	0.1769	0.889	0.8645	11.4153	28.5232	16.6796
FCT	0.3354	23.4457	0.0573	0.0211	0.8295	0.1356	23.8359	36.7186	23.3484
IS	0.1218	5.6817	0.0346	0.012	0.6511	0.1062	0.9847	5.8394	1.5568
ISOLET	0.9308	151.9821	0.1063	0.0373	1.0864	0.1419	82.7747	123.2756	59.5029
MNIST	0.3202	41.2678	0.1068	0.0231	0.8555	0.0598	53.722	129.9373	74.0810
ODR	0.3156	56.9395	0.0576	0.0175	0.8576	0.0549	51.6944	51.0083	26.0801
SPF	0.0576	3.5059	0.0285	0.0072	0.6918	0.0354	0.5598	1.1896	0.6515
Semeion	0.0607	2.5935	0.0384	0.0116	0.6849	0.0429	3.2673	4.2307	2.6795
Texture	0.2214	63.5331	0.0514	0.0158	0.7844	0.0703	8.3535	28.9052	20.1014
VS	0.0522	0.4591	0.0242	0.006	0.6647	0.0228	0.8366	1.2248	0.7616

## Data Availability

All the data in this paper are publicly available. They can be accessed at http://archive.ics.uci.edu/ml, http://www.vision.caltech.edu/feifeili/Datasets.htm, http://www.cs.nyu.edu/~roweis/data.html, and https://www.csie.ntu.edu.tw/~cjlin/libsvmtools/datasets/multiclass.html (all accessed on 10 April 2022).
